# Dynamic monitoring of rice plant height during the early growth stage using UAV-LiDAR and GWAS analysis of growth rate

**DOI:** 10.3389/fpls.2026.1795968

**Published:** 2026-06-10

**Authors:** Weicheng Xu, Fuyan He, Zhongyuan Chen, Guoyou Huang, Taotao Yang, Xiaozhe Bao, Longmei Wu, Wenzhe Liu, Feng Jiang, Junliang Zhao, Bin Zhang

**Affiliations:** 1 Rice Research Institute, Guangdong Academy of Agricultural Sciences, Guangzhou, China; 2 Huizhou Technician Institute, Huizhou, China; 3 Rice Research Institute, Guizhou Academy of Agricultural Sciences, Guizhou, Guiyang, China; 4 College of Agriculture & Biology, Zhongkai University of Agriculture and Engineering, Guangzhou, China

**Keywords:** GWAS, phenotype extraction, rice early growth vigor, rice plant height, UAV remote sensing

## Abstract

Plant height during the early growth stage of rice is a key indicator reflecting canopy establishment rate, tillering potential, and overall growth vigor, all of which critically determine final yield formation. Conventional manual measurements fail to capture high-frequency, continuous, and non-destructive monitoring of plant height dynamics, limiting the understanding of early growth vigor and its genetic mechanisms. In this study, a UAV-based LiDAR system was employed to acquire canopy point clouds of 211 rice accessions across ten time points within 40 days after transplanting. High-resolution canopy height models (CHMs) were generated, and continuous plant height trajectories H(t) were reconstructed using piecewise cubic Hermite interpolation (PCHIP). The first derivative V(t) quantified growth rate dynamics and identified the timing of maximum growth (T_max_), enabling precise differentiation of early growth patterns among geno-types. Genome-wide association analysis (GWAS) using a mixed linear model (MLM, Q+K) detected 604 significant SNPs, among which 33 were stably expressed across environments. Five candidate genes were identified within ±200 kb windows, mainly encoding proteins related to cell elongation, hormone signaling, and photosynthetic metabolism. The results highlight that LiDAR-based dynamic monitoring of plant height, coupled with genomic association analysis, provides a robust framework for quantifying rice early growth vigor and elucidating its molecular basis, offering valuable guidance for breeding high-vigor “early-establishing” rice cultivars.

## 1 Introduction

Rice (Oryza sativa L.), one of the most important staple crops worldwide, plays a vital role in global food security through its yield and quality. During the entire growth cycle of rice, the early growth stage represents a critical window that determines sub-sequent tillering capacity, the establishment of photosynthetic organs, root system development, and ultimately the yield potential of the crop (Mahender et al., 2015; Shi et al., 2020). At this stage, plants rapidly accumulate aboveground biomass and construct root architecture, thereby laying the material foundation for vigorous mid-stage tillering and later reproductive growth. A faster increase in plant height during the early growth period often indicates earlier canopy closure—which improves light interception efficiency—and the formation of a more developed root network, enhancing water and nutrient uptake. These advantages subsequently lead to stronger lodging resistance, greater biomass accumulation, and improved yield components such as effective panicle number and thousand-grain weight (Lin, 2015; Yuan et al., 2024). However, plant height assessment in rice has long relied on manual, periodic measurements, which suffer from high labor intensity, low spatiotemporal resolution (covering only limited sample sizes), and considerable subjectivity. Such limitations make it difficult to meet the demands of large-scale field production for high-throughput and continuous dynamic monitoring. Therefore, developing efficient and accurate non-contact approaches for monitoring early-stage plant height growth and elucidating the underlying genetic regulation is of great significance for promoting precision rice cultivation and molecular design breeding.

The integration of unmanned aerial vehicles (UAVs) with light detection and ranging (LiDAR) technology provides an innovative tool for dynamic phenotyping of crops during early growth stages (Balestra et al., 2024). LiDAR actively emits laser pulses and records the returned signals reflected from target surfaces, enabling the acquisition of highly ac-curate three-dimensional spatial coordinates with centimeter-level precision. When combined with the mobility and operational flexibility of UAV platforms, this system can rapidly generate high-resolution digital surface models (DSMs) and digital terrain models (DTMs) across large agricultural fields, thereby allowing non-contact and nondestructive estimation of crop height through DSM–DTM differencing. In agricultural applications, the three-dimensional structural information derived from LiDAR provides a more comprehensive representation of crop spatial characteristics than conventional two-dimensional imagery, enabling more efficient extraction of canopy height, density, inclination, and other structural attributes (Gu et al., 2020). Furthermore, the integration of UAV-based LiDAR point clouds with spectral data allows simultaneous characterization of both canopy spectral properties and three-dimensional structure, which significantly enhances canopy segmentation accuracy, variety classification performance, and community structural feature extraction. This fusion approach is particularly advantageous in complex canopy environments and fields containing multiple coexisting crop varieties (Li et al., 2025).

In recent years, UAV-mounted LiDAR systems have been increasingly applied to agricultural phenotyping, demonstrating high accuracy in estimating plant height, canopy structure, and biomass in crops such as maize, wheat, and rice. Ten Harkel et al (Ten Harkel et al., 2019). assessed UAV-LiDAR for estimating crop height and above-ground biomass for potato, sugar beet and winter wheat, showing good accuracy for height and biomass traits, confirming UAV-LiDAR’s potential for crop phenotyping. A UAV-LiDAR study on lodged maize used airborne LiDAR data to quantify structural changes and plant height under different stress conditions, illustrating applicability in cereals such as maize (Zhou et al., 2020). Gnädinger et al. further demonstrated that UAV-derived three-dimensional crop surface models are effective for characterizing spatial variability in maize height and biomass at the field scal (Gnädinger and Schmidhalter, 2017).

However, extracting plant height during the early growth stage differs substantially from that at maturity—when canopy structure is stable and height differences are pronounced. The challenges are mainly reflected in three aspects. First, sparsity and noise in early‐stage point clouds: at the seedling stage, plants are short and leaves are not fully expanded, resulting in limited LiDAR returns from canopy tops and strong interference from background surfaces such as water or soil (Tilly et al., 2014; Wang et al., 2024). This often leads to uneven point density, poor separability between ground and canopy peaks, and a high proportion of noise points caused by water‐surface reflections, weeds, or other disturbances (Holman et al., 2016; Song et al., 2025). Second, poorly defined canopy structure at the population level: early in development, small plant spacing and insufficient vertical stratification produce a relatively flat canopy surface, making canopy‐top identification based solely on local maxima unreliable. Consequently, statistical height metrics or surface‐modeling approaches are often required to characterize canopy height at the community scale (Bendig et al., 2013; Zhang et al., 2025). Third, temporal consistency under rapid dynamic growth: early‐stage plant height changes rapidly, and plant morphology evolves continuously, placing high demands on spatial–temporal registration accuracy, consistent plant identification, and stable tracking across multi‐temporal UAV‐LiDAR datasets. Without robust temporal alignment, errors in height estimation may propagate through the time series, compromising the reliability of growth rate and incremental change analyses (Rodriguez-Sanchez et al., 2024; Yang et al., 2024). Therefore, developing UAV–LiDAR point cloud processing algorithms tailored to the characteristics of early growth stages—including point cloud denoising, canopy-top detection, and temporal matching—is essential for achieving accurate monitoring of early-stage plant height growth dynamics.

In terms of elucidating the genetic basis, although traditional breeding practice has long recognized the importance of early vigor—characterized by rapid growth af-ter transplanting—for final yield, the genetic regulatory mechanisms underlying the early-stage plant height growth rate in rice remain largely unclear. Classical studies have primarily focused on plant height measured at a single time point at maturity, or on growth rates averaged across the entire growing season, while overlooking the dynamic growth processes occurring during the early developmental stage. During this period, plant growth depends mainly on nutrients stored in the seed and the limited nutrient uptake by the nascent root system; thus, height increase is governed by distinct genetic pathways associated with cell division, cell elongation, and meristem activity. YANG et al (Yang et al., 2024). reported that GW5/GSE5 (LOC_Os05g09520) influences early shoot dry weight and growth rate, revealing genetic regulatory pathways in the early growth phase that differ from those operating at maturity. ZHAO et al (Zhao et al., 2022). demonstrated that the miR528–D3 module negatively regulates plant height by modulating GA and ABA homeostasis, thereby affecting internode elongation and ultimately reducing plant height, adding a new regulatory layer to D3-mediated height control in rice. Genome-wide association study (GWAS) technology enables the identification of key genes or quantitative trait loci (QTLs) underlying early vigor—such as Os-CPS1—and facilitates the dissection of polygenic regulatory networks. These findings provide molecular tools for the targeted improvement of early vigor in breeding pro-grams, for example, selecting rice varieties with high growth rates during the first 40 days after transplanting to promote robust early seedling development (Dai et al., 2022; Ma et al., 2022).

In this study, 211 rice varieties were used to investigate the dynamic extraction of early-stage plant height. Multi-temporal UAV–LiDAR point cloud data were processed to obtain plant height at key developmental stages, and early growth parameters such as maximum growth rate and the time at which the peak growth rate occurs were de-rived based on height‐growth trajectories. Furthermore, genome-wide association analysis was conducted using whole-genome resequencing data to identify the genetic loci underlying early plant height growth rate. The objectives of this study were to:

1. Develop a point cloud denoising, individual-plant segmentation, and canopy height detection workflow suitable for early plant morphology, enabling high-throughput extraction of plant height across large and diverse germplasm populations;
2. Construct growth-rate curves based on time-series plant height, extract key parameters such as maximum growth rate and its occurrence time, and thereby enable quantitative comparisons of early vigor among different genotypes;
3. Integrate temporal phenotypes with resequencing data to identify QTLs and candidate genes controlling early plant height growth rate, providing insights for the genetic dissection and molecular improvement of early vigor.

In this study, we establish an integrated UAV-LiDAR–based phenotyping work-flow to quantify early-stage rice plant height dynamics and link temporal growth-rate traits with genome-wide association analysis. Rather than introducing new algorithms, this framework organizes existing phenotyping and genetic analysis methods into a unified, stage-specific analytical pipeline tailored to early growth vigor. These out-comes hold important theoretical and practical significance for improving rice yield stability and resource-use efficiency under variable environmental conditions.

## 2 Materials and methods

### 2.1 Software and environment configuration

All data processing and visualization analyses were conducted in a Python 3.9 environment on a Windows 10 platform. The main software and third-party libraries used in the workflow include:

1. Data preprocessing and spatial analysis:

Point cloud data stitching was performed using DJI Terra 1.1.0.12; rasterio 1.3+, geopandas 0.14+, shapely 2.0+, and laspy 2.4+ were used for LiDAR point cloud read-ing and writing, ground point filtering, vector clipping, and CHM calculation and rasterization.

2. Numerical computation and time-series modeling:

numpy 1.26+ and pandas 2.2+ were employed for matrix operations and batch statistical processing.

3. Signal smoothing and interpolation:

The signal.savgol_filter function in scipy 1.11+ was used to perform Savitzky–Golay smoothing, and PchipInterpolator was used to implement piecewise cubic Her-mite interpolation (PCHIP).

4. Visualization and plotting:

matplotlib 3.8+ and seaborn 0.13+ were used to generate plant height time-series curves, growth rate curves, and 3D CHM visualizations.

5. Scripting and version management:

All scripts were executed within an Anaconda (Python 3.9 base) environment, and Jupyter Notebook was used for workflow documentation and reproducibility verification.

### 2.2 Experimental design

**Figure 1:**
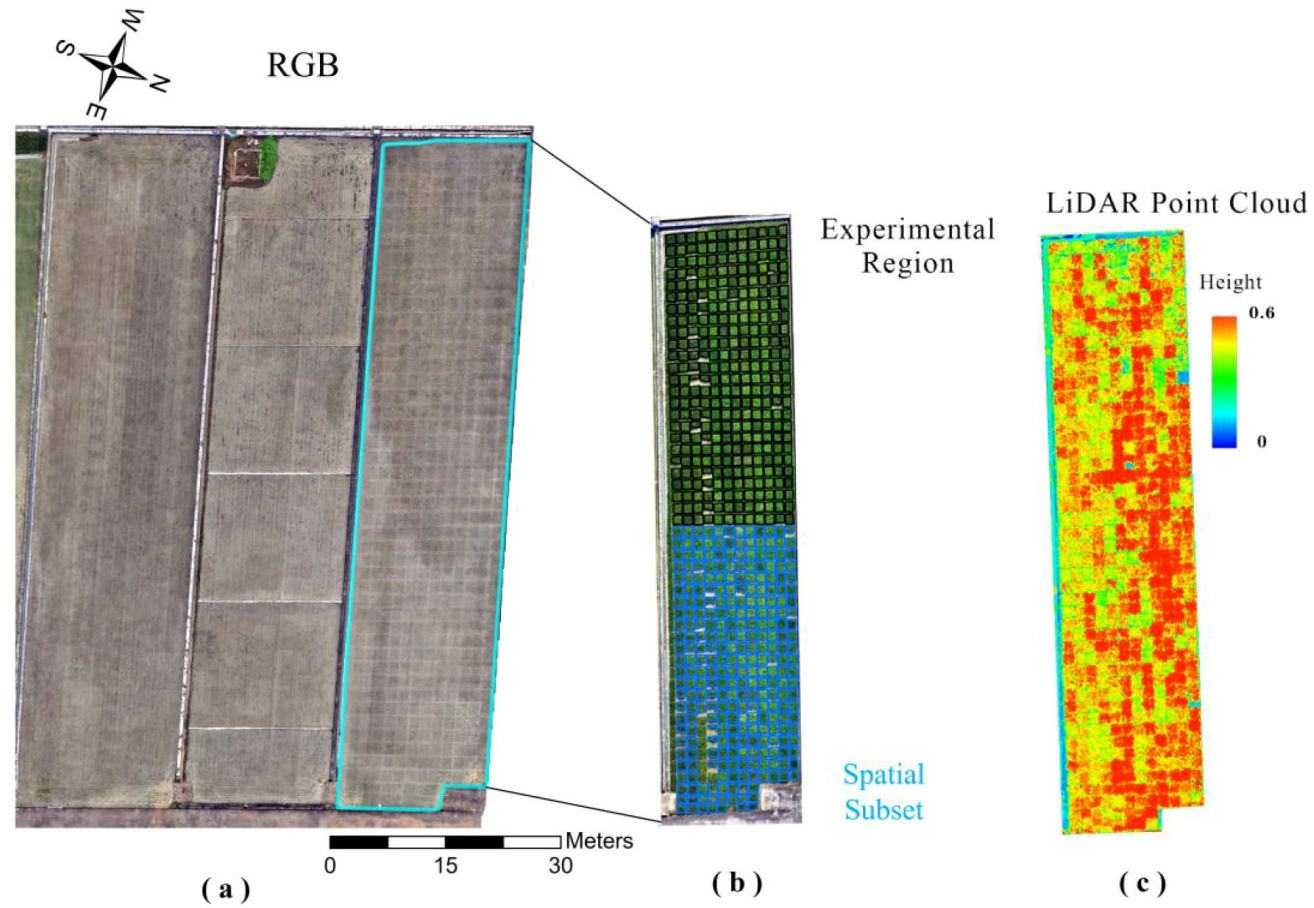
Experimental area and data: **(a)** RGB orthomosaic of the experimental area; **(b)** Plot layout of the experiment; **(c)** Point cloud data with elevation coloring.

The experimental site was located at the Baiyun Experimental Station of the Guangdong Academy of Agricultural Sciences in Guangzhou, Guangdong Province, China (113°25′42″E, 23°23′33″N) (**Figure 1A**), which is situated in a double-cropping rice production region. Early-season rice is typically planted from April to July, and late-season rice from August to November. To ensure diversity in the experimental materials, 135 international rice varieties originating from different regions of the world (provided by the International Rice Research Institute), along with 76 Guang-dong rice varieties bred across different decades (from the Rice Research Institute of the Guangdong Academy of Agricultural Sciences), were selected, totaling 211 varieties. Each variety was planted in two independent plots within a single experimental block, resulting in 422 plots in total. Each plot contained only one rice variety, and no mixed planting of varieties was conducted within any plot. Since the specific variety identity was not the focus of this study, individual variety names are not listed here.

Each plot contained 64 plants arranged in 8 rows × 8 columns. Rice seedlings were transplanted manually following local conventional practices. Transplanting positions were guided using a positioning rope with fixed knot intervals, resulting in a row spacing of 6 cun (19.98 cm) and a plant spacing of 5 cun (16.65 cm), where cun is a commonly used traditional length unit in Chinese farmland measurements. Before transplanting, knot positions along the rope were adjusted using a measuring tape to ensure consistent spacing across all plots (Keightley, 1995). Cultivation practices followed local conventional management. Fertilizer inputs were standardized on an area basis and expressed as application rates per hectare. The fertilization regime consisted of a basal application equivalent to 147 kg·ha^−1^ urea, 90 kg·ha^−1^ potassium chloride (KCl), and 390 kg·ha^−1^ superphosphate, followed by additional nitrogen and potassium inputs during vegetative and reproductive stages. Specifically, 88 kg·ha^−1^ urea and 90 kg·ha^−1^ KCl were applied during the tillering phase, and 59 kg·ha^−1^ urea together with 120 kg·ha^−1^ KCl were supplied at the panicle initiation stage. No phosphorus fertilizer was applied beyond the basal dressing. Data were collected in both the early and late sea-sons of 2025. The transplanting date for the early-season rice was April 4, 2025, and for the late-season rice was August 9, 2025. UAV–LiDAR data (**Figure 1C**) were acquired every 4 days within the first 40 days after transplanting, resulting in 10 acquisition periods per season. Plant height was manually measured for 3 representative plants at May 11 and August 30 and used to validate the accuracy of subsequent plant height monitoring. Although only one physical replicate block was established per season, environmental robustness was enhanced by conducting the analysis across four environmental conditions (early/late season × experimental/repeat plots), enabling cross-environment validation of phenotypic patterns. Within each seasonal experiment, spatial sub-regions were established under different nitrogen management regimes. The main experimental region received conventional nitrogen fertilization, whereas an adjacent spatial sub-region was subjected to a zero-nitrogen (N0) treatment. This experiment was conducted within a single calendar year (2025) at one location, the terms “spatial subset” and “late rice spatial subset” refer to spatial partitions within the same block rather than independent biological replicates. Therefore, genotype effects cannot be fully disentangled from random environmental fluctuations, and genotype-by-environment interactions cannot be explicitly modeled.

### 2.3 Drone cloud point data acquisition

Point cloud data were acquired using a DJI M350 RTK UAV equipped with a ZENMUSE L2 LiDAR system. This integrated system combines a Livox LiDAR module, a high-precision inertial measurement unit (IMU), a differential positioning module (RTK), a mapping camera, and a three-axis gimbal. The detailed hardware specifications are provided in **Table 1**. Owing to the onboard RTK module, centimeter-level point cloud accuracy can be achieved even in the absence of ground control points.

**Table 1:** Point cloud data collection hardware parameters.

Parameter and specification	ZENMUSE L2
LiDAR	
Ranging accuracy	20mm at 20m
Measurement	450 meters (reflectivity 50%, 0 klx)
Maximum number of echoes supported	5
Field of View (FOV)	Repeat scanning: 70°× 3°
Data acquisition rate	Single echo: 240000 points/s Multiecho: 1200000 points/s
Laser wavelength	905nm
Scanning frequency	240kHz
Laser beam footprint at 20 m	24mm× 8mm
IMU(Inertial Measurement Unit)	
Refresh rate	200Hz
Course Accuracy; Pitch accuracy	0.15°; 0.025°
RGB Camera	
Resolution	5280×3956
Focal length	8.8mm/24mm (Equivalent full frame)
Aperture	f/2.8 - f/11

During data collection, to balance acquisition efficiency and data accuracy, we set the UAV flight speed to 0.7 m/s and the flight altitude to 20 m, resulting in a point density of 70,452 points/m². The accompanying software DJI Terra (version 4.1.0, DJI Innovations Co., Ltd., Shenzhen, China) was used to read the LiDAR point cloud, IMU records, flight trajectory files, and other relevant data. The software performed POS processing, point cloud coloring, and stitching. The final outputs were LAS format point cloud files containing the three-dimensional coordinates of each point, scan an-gle, return number, pulse intensity, and GPS timestamp.

### 2.4 Point cloud data processing

**Figure 2:**
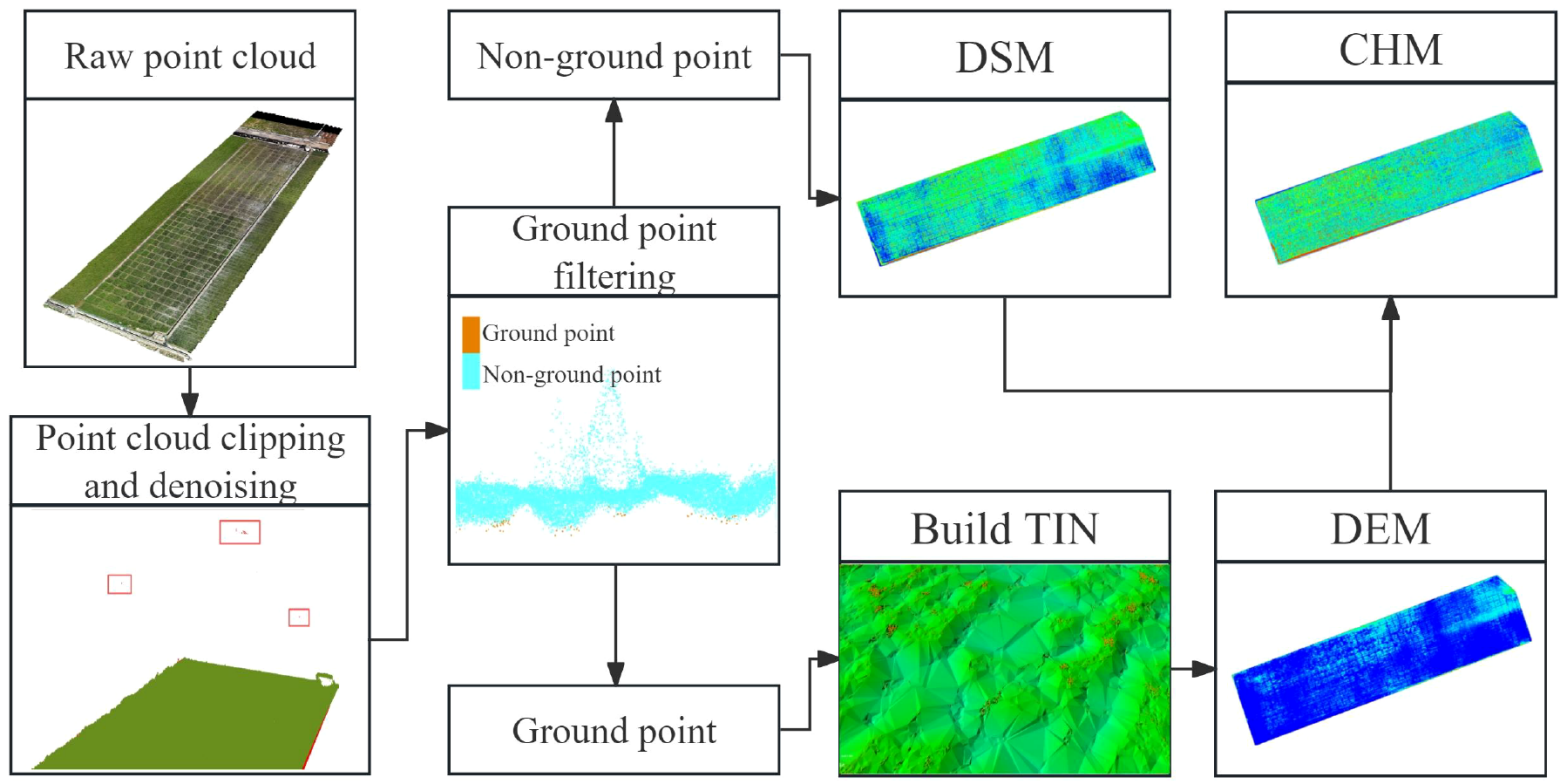
Point cloud data processing flow.

To ensure the accuracy of subsequent plant height extraction and dynamic change analysis, the raw point cloud data must undergo systematic preprocessing, including noise removal, ground point filtering, and rasterization, to generate high-quality digital surface models (DSM), digital terrain models (DEM), and canopy height models (CHM). This workflow consists of five major steps: noise point removal, ground point classification, construction of a triangulated irregular network (TIN), rasterization, and canopy height calculation (**Figure 2**).

#### 2.4.1 Point cloud denoising

**Figure 3:**
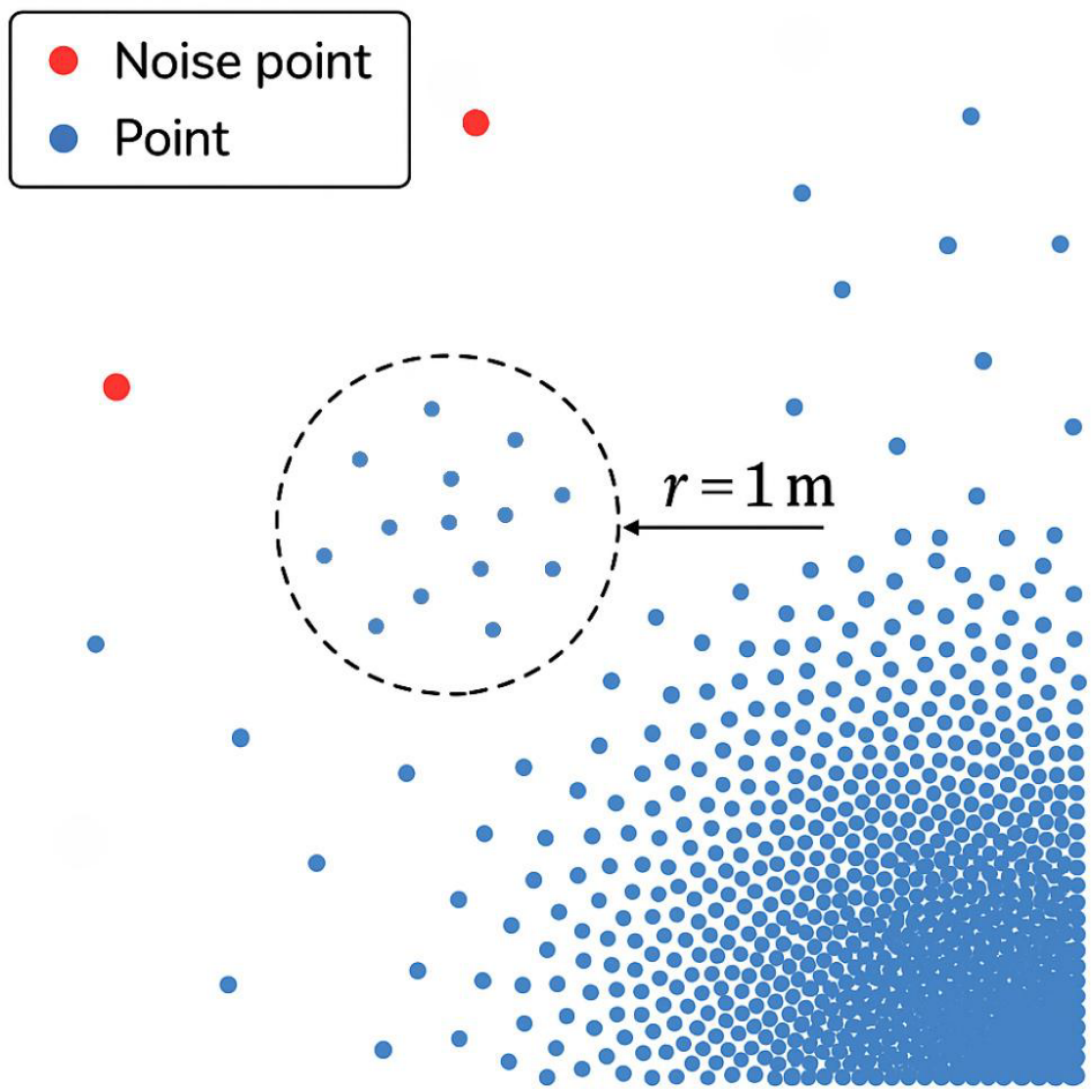
Radius-based filtering.

Due to system errors and random disturbances arising from environmental factors, isolated points and elevation outliers are inevitably present in the raw point cloud. These abnormal points can cause elevation artifacts in the subsequent digital surface model (DSM), thereby affecting the accuracy of plant height extraction. In this study, a radius-based filtering algorithm was employed to identify and remove isolated points. Specifically, for each point, the number of neighboring points within a predefined radius was calculated; points with fewer neighbors than the specified threshold were classified as noise (**Figure 3**). Based on the density of the point cloud acquired in this study, points with fewer than 10,000 neighbors within a 1-m radius were considered noise.

#### 2.4.2 Ground point filtering

This study employed the Progressive TIN Densification (PTD) algorithm for ground filtering. In the filtering algorithm tests organized by the International Society for Photogrammetry and Remote Sensing (ISPRS), the PTD algorithm achieved the best overall performance. The core idea of PTD is to first construct an initial Triangulated Irregular Network (TIN) using selected ground seed points. The remaining points are then iteratively evaluated, and points that meet the criteria are added to the ground point set, allowing the TIN to become increasingly refined in subsequent iterations. This iterative process continues until no new ground points are detected or the maxi-mum number of iterations is reached.

The algorithm is implemented through the following steps:

1. Ground seed point selection is performed first. A cuboid bounding box is de-fined to encapsulate the point cloud, which is then divided into n rows and m columns. Based on the principle of selecting the lowest local elevation, ground seed points are uniformly extracted from the original point cloud, while the remaining points are treated as candidate points for evaluation. The values of n and m are calculated as follows (Equation 1):

(1) $$\{\begin{array}{c} n=\left[\frac{x}{b_{\max}}\right]\\ m=\left[\frac{y}{b_{\max}}\right] \end{array}$$

where x and y represent the length and width of the cuboid boundary, respectively, and b_max_ is set to the ridge width in this study.

**Figure 4:**
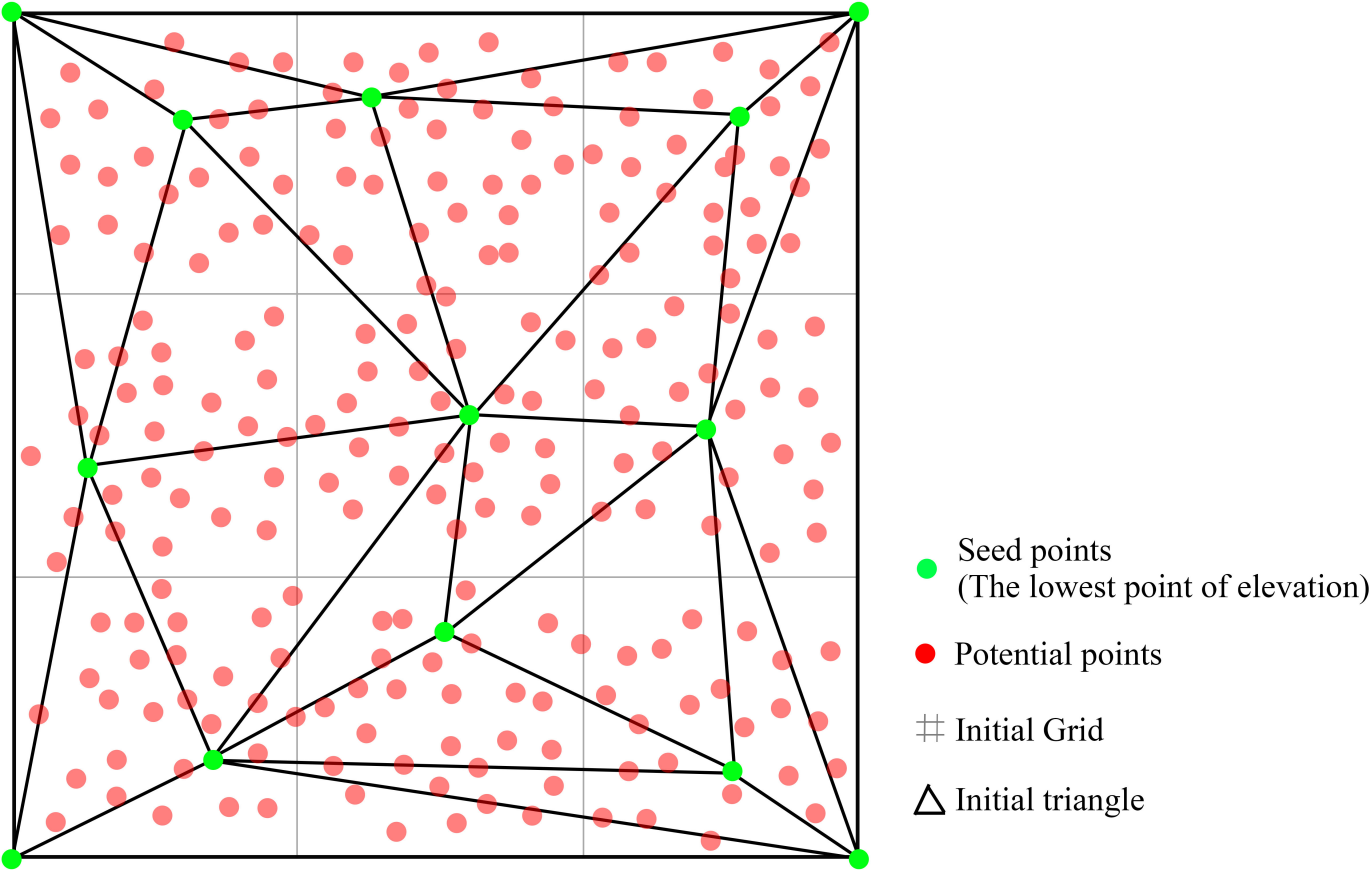
Point cloud data processing flow.

Subsequently, the initial ground seed points are used to construct the initial TIN (**Figure 4**).

**Figure 5:**
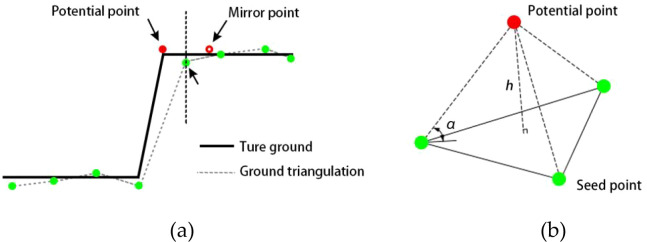
Iterative encryption process :**(a)** Mirroring process and **(b)** Calculation of α and h.

2. Ground points are iteratively densified from the candidate points. For each candidate point, its projected coordinates on the x–y plane are used to identify the corresponding triangular facet in the TIN, and the slope of that facet is computed. If the slope exceeds the predefined maximum slope α1, a mirror point is used for classification (**Figure 5A**). The candidate point is then assigned the same class as its mirror point. Specifically, for a candidate point P(xP,yP,zP), the vertex with the highest elevation in its corresponding triangle, denoted as Ph(xn,yn,zn), is located. The coordinates of mirror point P are calculated as follows (Equation 2):

(2) $$\{\begin{array}{l} x_{mirror}=2x_{\text{n}}-x_{\text{p}}\\ y_{mirror}=2y_{\text{n}}-y_{\text{p}}\\ z_{mirror}=2z_{\text{p}} \end{array}$$

3. The parameter α1 represents the maximum allowable terrain slope threshold, which controls the tolerance for local surface inclination during ground point classification. It was empirically determined based on field topography and point cloud density to balance ground preservation and vegetation removal.

If the slope of the triangular surface is less than the maximum slope threshold α1, the vertical distance h between the candidate point and the triangular surface is calculated. In addition, the angle α between the line connecting the candidate point to the nearest triangle vertex and the triangular plane is computed (**Figure 5B**). If both conditions α<α1 and h<h1 are satisfied—where h1 denotes the maximum allowable elevation difference—the candidate point is classified as a ground point and added to the ground point set. The TIN is then reconstructed, and the above procedure is iteratively repeated until no new ground points are identified or the maximum number of iterations is reached.

#### 2.4.3 Canopy height model generation

To extract spatial information on terrain and canopy structure from the point cloud data, a Digital Surface Model (DSM) and a Digital Elevation Model (DEM) were constructed separately. The DSM represents height information of the ground surface as well as vegetation, buildings, and other aboveground features, whereas the DEM describes only the underlying terrain undulation. The Canopy Height Model (CHM), calculated as the difference between the DSM and DEM, is fundamentally derived from these two models.

For DSM construction, this study employed the Inverse Distance Weighted (IDW) interpolation method (Moussa and Abboud, 2024). This approach assumes a distance-decay effect in elevation values, meaning that samples closer to the interpolation location exert a stronger influence than those farther away. The specific calculation formula is as follows (Equation 3):

(3) $$Z\left(x_{0},y_{0}\right)=\frac{\sum_{i=1}^{n}\frac{Z_{i}}{d_{i}^{2}}}{\sum_{i=1}^{n}\frac{1}{d_{i}^{p}}}$$

where Z(x0,y0) is the interpolated elevation value, Zi is the elevation of the i-th sample point, di is the distance between the interpolation point and the sample point, and p is the distance decay exponent (set to 2 in this study).

This method preserves canopy structural details while smoothing local height fluctuations, enabling the DSM to achieve good continuity and accuracy in representing rice canopy height.

The DEM was constructed using a Triangulated Irregular Network (TIN) interpolation approach based on ground points. Using the three-dimensional coordinates of the ground points, a TIN was generated through Delaunay triangulation —a widely used triangulation method that constructs a mesh of non−overlapping triangles by connecting points such that no other point lies inside the circumcircle of any triangle, which tends to maximize the minimum angle of triangles and avoid elongated, unstable elements, forming an irregular mesh surface (Adedapo and Zurqani, 2024; Zheng et al., 2024). This allows precise characterization of subtle terrain variations and field ridge boundaries in paddy fields. The method provides stable fitting performance in environments with minimal terrain relief, avoiding the terrain distortions that may be introduced by overly smoothed interpolation methods.

Both the DSM and DEM were exported at a spatial resolution of 0.01 m and projected to the WGS84/UTM Zone 49N coordinate system, consistent with the point cloud data. The canopy height model (CHM) was then obtained through pixel-wise subtraction of the DEM from the DSM. The resulting CHM clearly reflects height differences and spatial distribution patterns of rice canopy structure across experimental plots.

### 2.5 Analysis of dynamic changes in plant height

#### 2.5.1 Time series plant height extraction

**Figure 6:**
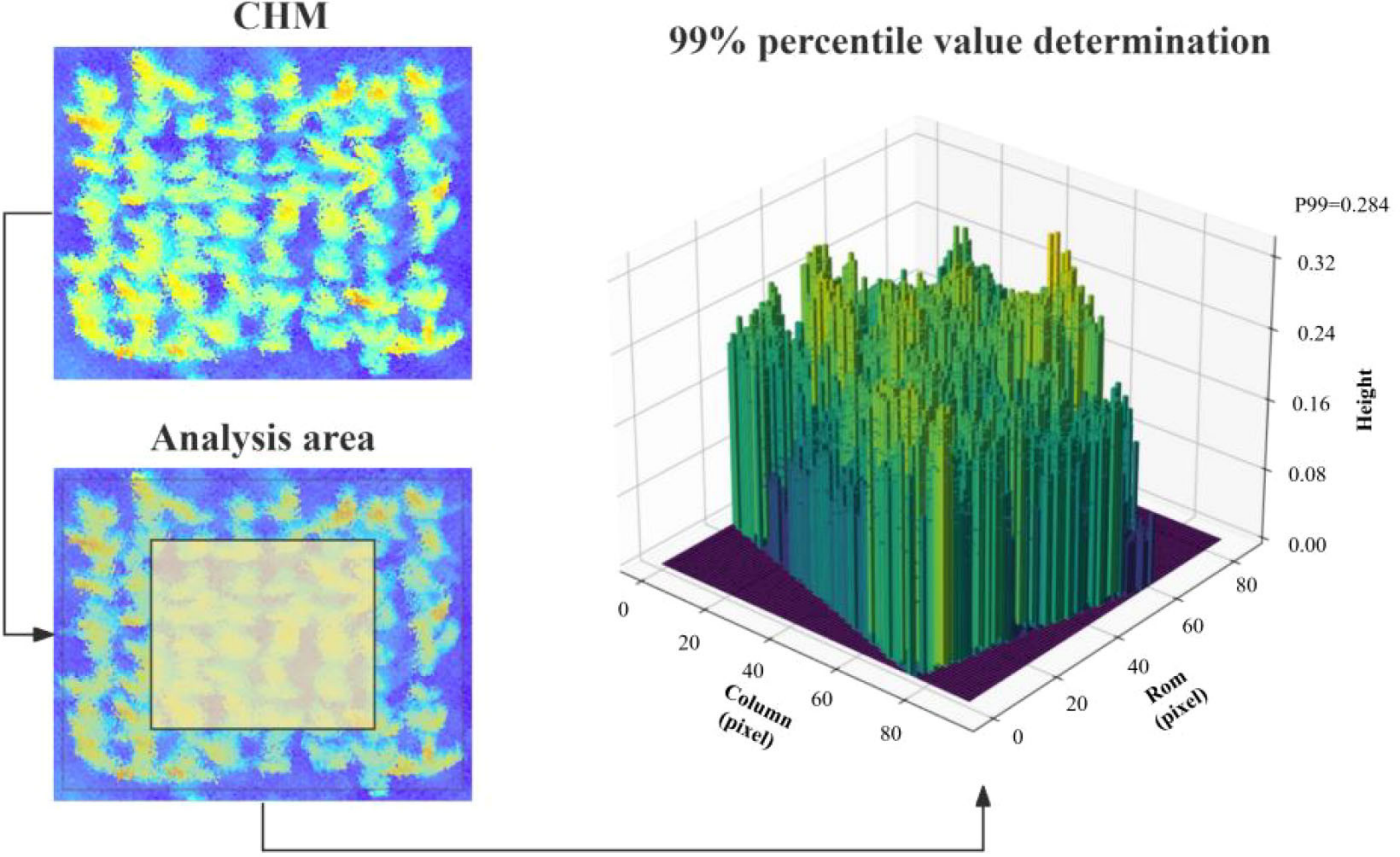
Plant height extraction process.

To achieve quantitative extraction of rice plant height time series, the canopy height model (CHM) generated in Section 2.3.3 was first spatially clipped using manually delineated plot boundary vector files (Shapefile format) corresponding to the experimental design. To reduce boundary effects, a central 5 × 5 grid region was selected within each plot as the analysis unit, ensuring that all units had equal area. Each plot corresponds to a specific variety (including replicates). Through vector masking, the set of CHM pixel values within each plot was extracted, and the 99th percentile (P99) of CHM pixel height was calculated as the plant height of that plot. The overall work-flow is shown in **Figure 6**.

Compared with P99, P90 and P95 tend to underestimate canopy height during the seedling stage due to the influence of locally depressed leaves and ground returns. In contrast, P99 effectively captures the uppermost canopy layer during periods of dense canopy cover. Therefore, under the canopy structural conditions and point cloud density of this study, using P99 more accurately represents the top canopy structure and avoids the underestimation that can arise when mean values are biased by noise or ground points (Luo et al., 2021).

Finally, plant height time series from t1 to t10 were obtained for each variety and used for subsequent growth dynamics analysis. By sequentially clipping and statistically analyzing the CHM data from the first to the tenth observation period (t1–t10) after transplanting, a complete plant height time series was generated for each variety.

#### 2.5.2 Dynamic fitting of plant height

To characterize the early-stage plant height dynamics of different rice varieties, a time-series analysis workflow was developed based on multi-temporal canopy height data extracted from UAV-LiDAR. The workflow consists of smoothing preprocessing, interpolation modeling, derivative computation, and peak extraction.

First, noise evaluation was performed on the multi-temporal plant height series for each variety. To suppress measurement errors that may affect derivative calculations, a Savitzky–Golay smoothing filter was applied for mild smoothing, and amplitude–duration joint thresholding was implemented to remove potential adjacent spurious peaks in the derivative curve. Subsequently, piecewise cubic Hermite interpolation (PCHIP) was used to interpolate the plant height series—originally acquired every 4 days—to a daily temporal resolution. PCHIP preserves local monotonicity and shape without introducing overshoot, thereby yielding a continuous function H(t) that rep-resents plant height dynamics over time. Similarly, piecewise interpolation methods such as piecewise cubic Hermite interpolation (PCHIP) are commonly used in environmental and ecological time series for reconstructing daily trajectories from discretely sampled data while preserving monotonicity and avoiding overshoot—an important property when estimating growth curves (Dronova et al., 2022; Wang X. et al., 2022).

Based on the continuous function H(t), an analytical derivative was computed to obtain the growth rate curve V(t)=dH/dt. The maximum value of V(t) within the open interval (t_min_,t_max_) was identified to determine the peak growth rate V_max_ and its corresponding time T_max_, which characterize the highest early-stage growth rate and the timing at which it occurs, respectively.

Given that the V(t) curve may exhibit multiple peaks, a two-step filtering strategy was adopted for peak extraction (Ge et al., 2021). First, local maxima were detected to obtain candidate peaks. Then, based on peak morphology—specifically, requiring that the descending magnitude on both sides of a peak be no less than 20%—noise-induced sharp spikes were removed. Only the primary peak reflecting physiological stage transitions was retained as T_max_. This strategy effectively avoids false peak identification caused by local data fluctuations (Kong et al., 2022).

This growth-rate parameterization transforms static height measurements into dynamic temporal traits, enabling subsequent genetic analysis to focus on growth timing rather than absolute plant stature.

#### 2.5.3 Error propagation control from height estimation to growth-rate extraction

UAV-LiDAR–derived plant height at the early growth stage is subject to measurement uncertainty caused by sparse canopy structure, background interference, and partial laser penetration. If not properly controlled, such uncertainty may be amplified during temporal differentiation and lead to unstable growth-rate estimation.

To mitigate error propagation, a three-stage constraint strategy was implemented before extracting growth-rate parameters. First, a Savitzky–Golay filter was applied to the raw multi-temporal height series to suppress high-frequency noise while preserving overall growth trends. Second, piecewise cubic Hermite interpolating polynomial (PCHIP) was used to interpolate the height series to daily resolution. Unlike high-order polynomial fitting, PCHIP preserves monotonicity and prevents artificial overshooting, ensuring physiologically plausible growth trajectories. Third, growth-rate peaks were screened using morphology-based criteria, requiring sufficient peak width and symmetric decline on both sides to exclude noise-induced spikes.

Through these constraints, the extracted timing of maximum growth rate (T_max_) reflects stable growth-stage transitions rather than local measurement fluctuations, providing a robust phenotype for subsequent GWAS analysis.

### 2.6 Genomic data and GWAS analysis

#### 2.6.1 Dynamic fitting of plant height

This study utilized genomic data from 211 rice accessions, all of which underwent whole-genome resequencing with an average sequencing depth of no less than 10× (where “×” denotes sequencing coverage, meaning the average number of times each nucleotide is read). To ensure the validity of the association analysis, quality control was performed on the raw variant data by removing loci with insufficient sequencing depth, filtering out rare variants with a minor allele frequency (MAF) less than 0.05, and excluding loci with a sample missing rate greater than 0.1. A total of 5,090,911 SNP markers were retained for subsequent association analysis.

#### 2.6.2 Phenotypic data and GWAS model

Early-stage plant height variation was derived from multi-temporal UAV–LiDAR point cloud data. Based on the time-series curves of individual plant height, the growth rate trajectory was obtained by differentiation, and the timing of the peak growth rate (T_max_) for each accession was identified as the phenotypic trait. This trait reflects differences in early growth rhythms among genotypes.

Genome-wide association analysis (GWAS) was conducted using a mixed linear model (MLM, Q+K), expressed as (Equation 4):

(4) Y=Xβ+Qγ+Kμ+ϵ

where Y is the phenotypic vector of the timing of peak growth rate, X is the geno-type matrix, β represents the fixed effect of each SNP on the trait, Q is the population structure matrix with its corresponding effect representing the influence of population structure on the phenotype, K is the kinship matrix with its associated random effect capturing the genetic covariance among individuals due to kinship, and ϵ is the normally distributed random error term.

The MLM framework effectively reduces false-positive associations caused by population structure and kinship. Due to the lack of multi-year and multi-replicate data, variance components and trait heritability were not estimated in this study.

#### 2.6.3 Significant threshold and candidate gene screening

GWAS was performed using the mixed linear model (MLM) implemented in the TASSEL 5.0 software package. Given that this study involved more than five million high-density SNP markers, applying a Bonferroni correction (approximately P< 1 × 10^−7^) would likely eliminate many true-effect loci due to its excessive stringency. Therefore, a more relaxed significance threshold of P< 1 × 10^−4^ was adopted for initial screening, followed by multi-environment co-localization (signals appearing in no fewer than three environments) as a robustness criterion, only association signals consistently detected in at least three environments were retained as stable QTLs. This conservative cross-environment filtering strategy effectively reduces environment-driven false positives and partially compensates for the limited number of physical field replicates. This two-step strategy ensures that the identified association signals are reliable, stable, and biologically meaningful. The term “multi-environment” in this study refers to seasonal and spatial conditions within the same year, rather than independent multi-year trials. Therefore, QTL stability across years and climatic variability cannot be inferred from the current dataset.

For SNPs surpassing the significance threshold, their chromosomal positions and physical coordinates were extracted. Candidate gene identification was conducted within a ±200 kb window (1 kb = 1,000 base pairs) centered on each significant SNP. Using the Osa1R7 rice genome database (https://rice.uga.edu/download_osa1r7.shtml) as a reference, all genes located within the QTL region and the surrounding 200 kb interval were retrieved to identify potential candidate genes associated with rice plant height. Spatial subect were not treated as independent environments in the GWAS model and were not used to inflate sample size or degrees of freedom.

## 3 Results

### 3.1 Verification of monitoring accuracy for plant height

**Figure 7:**
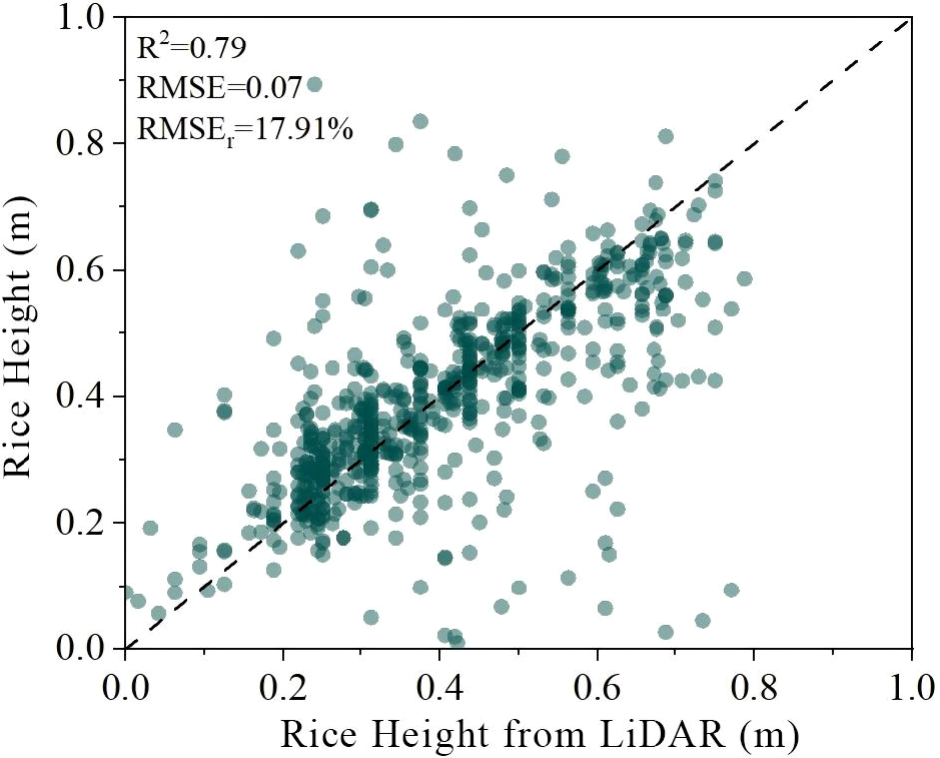
Comparison between manually measured plant height and canopy height models extracted from UAV-LiDAR.

To ensure the reliability of the population-level plant height time-series curves H(t) and their derivatives V(t) in this study, the accuracy of the underlying data source must first be validated. Field-measured plant height data were used as a reference to assess the accuracy of UAV-derived height estimates. Specifically, plant height measurements obtained from manual field surveys on May 11 and August 30 were com-pared with canopy height values extracted from the UAV LiDAR–derived Canopy Height Model (CHM) (**Figure 7**). The results showed that CHM-derived plant height values exhibited strong agreement with ground truth measurements, with a coefficient of determination (R^2^) of 0.79, a root mean square error (RMSE) of 0.07, and a relative RMSE (RMSEr) of 17.91%. While absolute height estimation may contain fixed bias at the seedling stage, the relative temporal pattern of height change within each variety remains stable, supporting the reliability of time-derived growth parameters.

### 3.2 Characteristics of dynamic changes in plant height

#### 3.2.1 Trend of plant height variation

**Figure 8:**
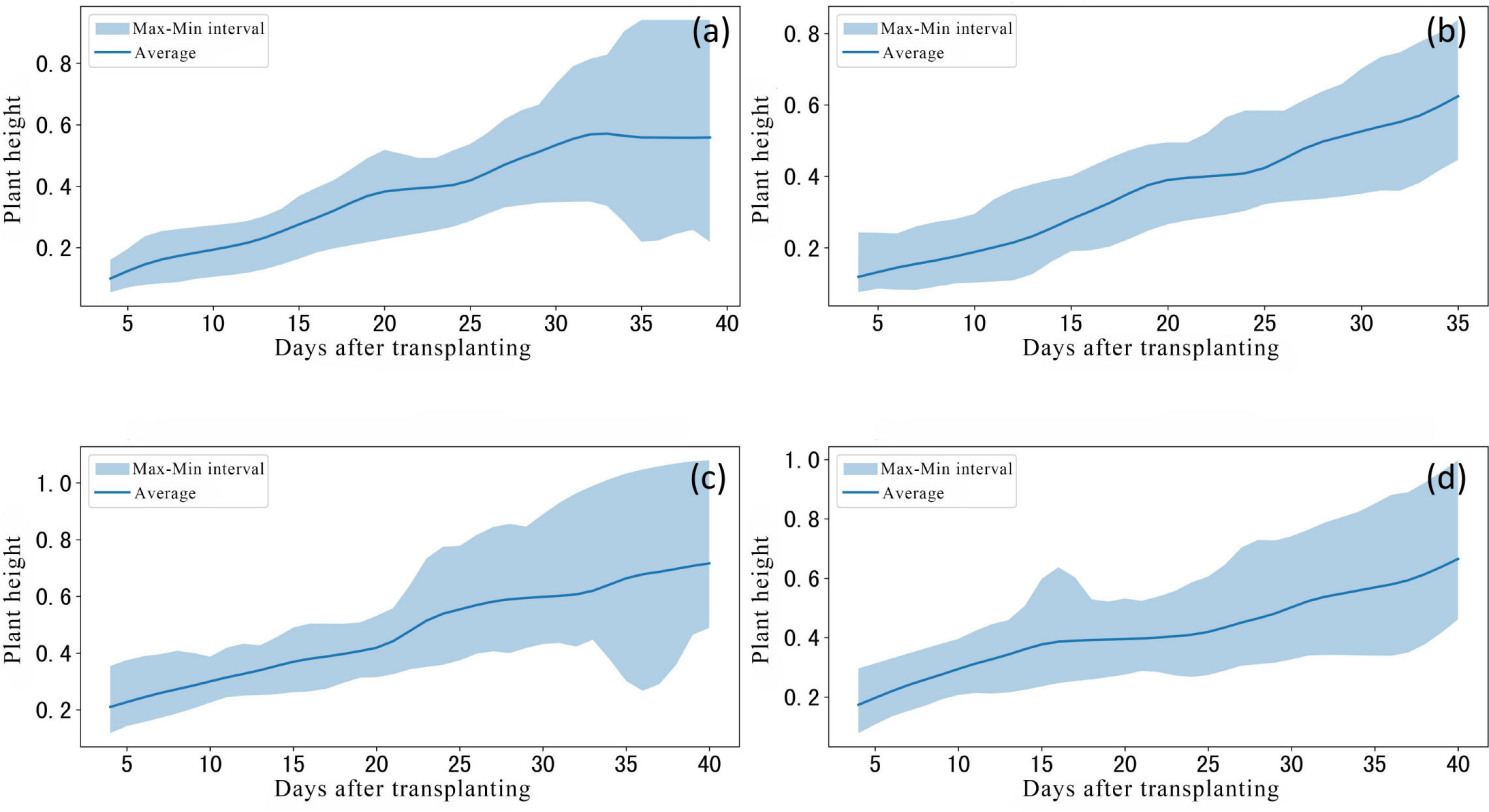
Population mean plant height curve and confidence intervals. **(a)** Early rice experimental region; **(b)** Early rice spatial subset; **(c)** Late rice experimental region; **(d)** Late rice spatial subset.

To gain deeper insight into the early-stage growth performance of different rice varieties after transplanting, this study utilized UAV LiDAR–derived population plant height data and applied PCHIP interpolation to the height measurements collected every four days. This produced a continuous and smooth population-level plant height curve H(t). By plotting the minimum–maximum range at each time point, the degree of height variation within the population was also illustrated. **Figure 8** presents the mean plant height curves H(t) and the corresponding minimum–maximum intervals for both early-season and late-season rice experimental plots, including their replicates.

Overall, plant height increased steadily with days after transplanting across all plots. However, clear differentiation emerged among the materials in terms of growth rhythm and final plant height. Notable differences were observed in initial height at the early growth stage, the timing of accelerated growth, and the ultimate height achieved by different varieties. During the earliest stages, plant height differences among varieties were relatively small, reflected in narrow intervals, indicating that growth trajectories were largely synchronized shortly after transplanting. As plants entered the rapid elongation phase, substantial variation appeared in growth rate and final plant stature, causing the intervals to widen progressively. Although the average growth trend eventually leveled off in some plots, inter-varietal height differences continued to expand, suggesting systematic genetic differences in developmental rhythm and final canopy architecture.

#### 3.2.2 Dynamic changes and peak analysis of plant height growth rate

**Figure 9:**
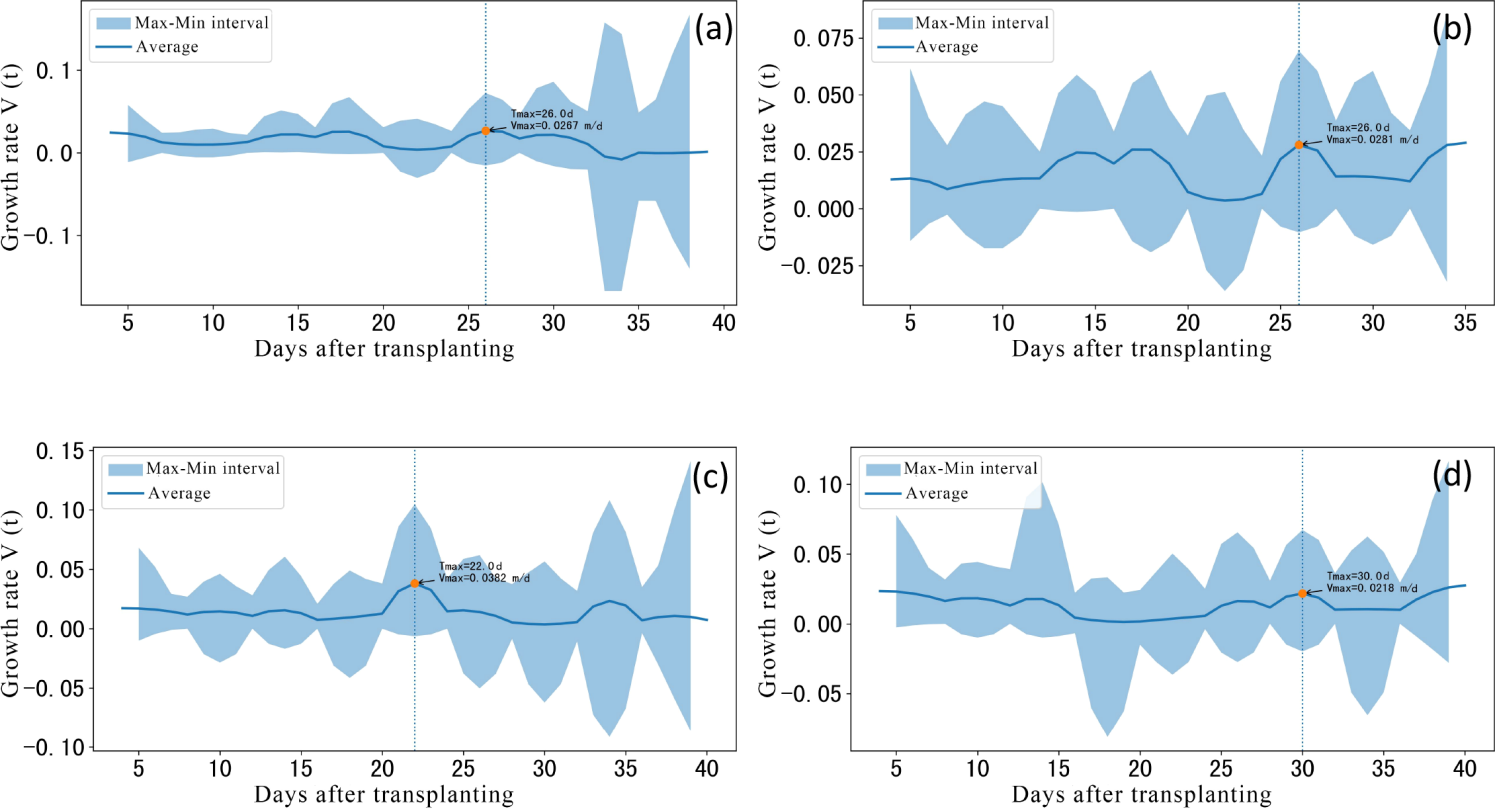
Population mean plant height growth rate curve and confidence intervals. **(a)** Early rice experimental region; **(b)** Early rice spatial subset; **(c)** Late rice experimental region; **(d)** Late rice spatial subset.

This study calculated the first derivative V(t) of the daily-fitted average plant height curve H(t) to reflect the growth rate dynamics of rice across different growth stages (**Figure 9**). The curve exhibited frequent fluctuations and complex morphology, indicating that growth rates were influenced by multiple interacting factors. Most populations showed multiple peaks in growth rates between 10–30 days after transplanting, with no distinct single dominant peak but rather a series of localized fluctuations. In the early rice experimental plots, the growth rate curves of both the test and replicate groups displayed multiple peaks, with the maximum rate (0.0382 m/d) occurring around day 22 in the test group. In contrast, the replicate group exhibited a delayed and less pronounced peak (0.0218 m/d) at day 30, suggesting a later onset of rapid growth potentially due to nitrogen deficiency in the replicate plots during the late rice season, This pattern is consistent with the well-established effects of nitrogen deficiency on delayed canopy development and suppressed early growth vigor. Notably, the “minimum-maximum range” of growth rates widened progressively over time, particularly after 25 days, with some groups showing intense fluctuations. This widening disparity highlights significant genetic heterogeneity in growth rates among cultivars. The findings imply that even when average growth rates appear stable, populations may harbor both early-maturing and late-maturing varieties, amplifying overall variability in growth kinetics. Notably, pronounced differences in both the magnitude and timing of peak growth rate were observed between spatial sub-regions within the same experimental block. These differences likely reflect systematic environmental or management heterogeneity rather than random noise or measurement error.

### 3.3 Differences in early growth dynamics between international and Guangdong rice varieties

**Figure 10:**
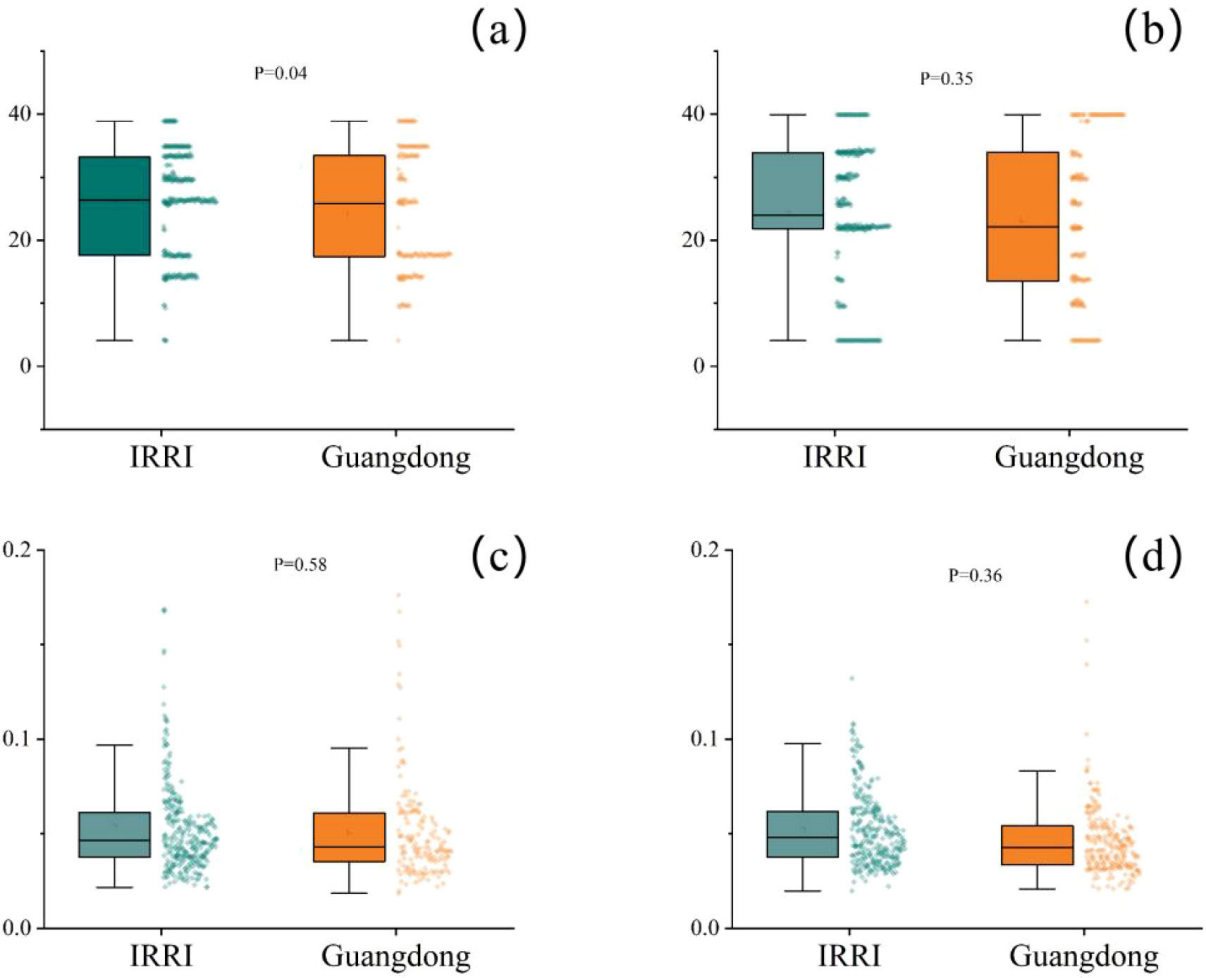
Comparison of early growth dynamics between international rice varieties (IRRI) and Guangdong local varieties. **(a)** Comparison of the timing of maximum growth rate (T_max_) for early-season rice; **(b)** Comparison of the timing of maximum growth rate(T_max_) for late-season rice; **(c)** Comparison of the maximum growth rate(V_max_) for early-season rice; **(d)** Comparison of the maximum growth rate(V_max_) for late-season rice.

Significant differences were observed in the timing of the maximum growth rate (T_max_) between international (IRRI) and Guangdong rice varieties (**Figure 10**). For early-season rice, Guangdong varieties reached T_max_ significantly earlier than IRRI varieties (P = 0.04), indicating faster early growth dynamics. A similar trend was observed for late-season rice, where Guangdong varieties also tended to reach T_max_ earlier; however, this difference did not reach statistical significance (P = 0.35).In contrast, no significant differences were detected in the maximum growth rate (V_max_) between IRRI and Guangdong varieties in either early-season or late-season rice (P = 0.58 and P = 0.36, respectively). This suggests that although the timing of peak growth differs be-tween the two groups, the magnitude of the maximum growth rate is largely comparable.

### 3.4 Plant height growth rate GWAS analysis

**Figure 11:**
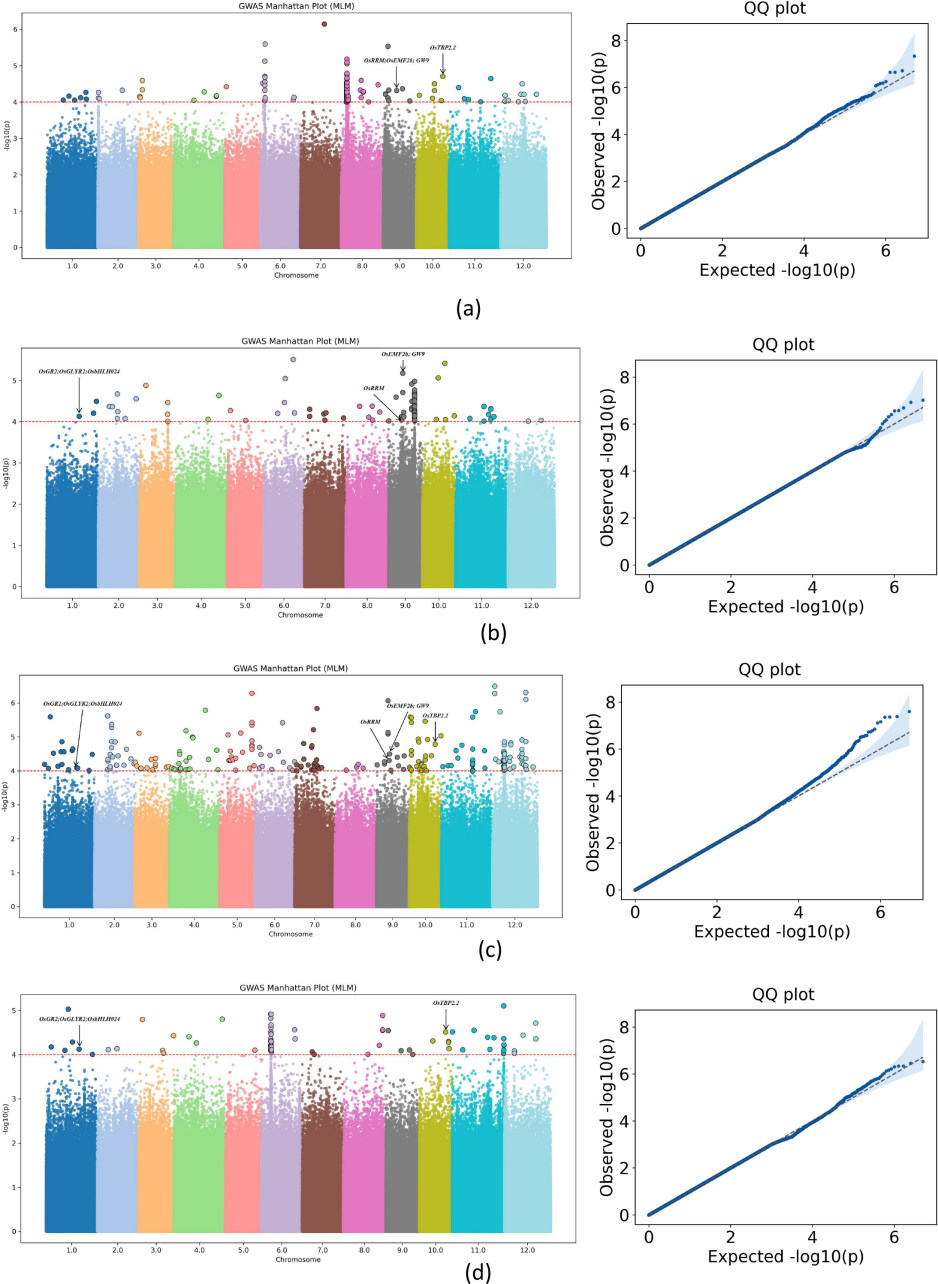
Manhattan and QQ mapping of QTL loci for the fastest early plant height growth rate in four rice growth conditions. **(a)** Early rice experimental region; **(b)** Early rice spatial subset; **(c)** Late rice experimental region; **(d)** Late rice spatial subset.

Using the 200 kb regions flanking the QTL-associated SNPs (upstream and down-stream) as the QTL intervals, genome-wide association studies (GWAS) were con-ducted on heading date-related traits across four environments. A total of 604 significantly associated QTL loci were detected, among which 33 loci (showing high environmental stability) were replicated in at least three environments. These stable signals were primarily distributed on chromosomes 1, 9, and 10, suggesting multi-chromosomal regulation of heading date variation. In early rice experimental and replicate plots, a distinct peak was observed near the 39 Mb region (1 Mb = 1 million base pairs) on chromosome 1, with good fit between observed and expected values in the QQ plot (**Figures 11A–D**), confirming the reliability of the associations. In late rice trials and replicates, stable significant intervals were detected on chromosomes 9 and 10, corresponding to candidate genes *OsRRM*, *OsEMF2b*, and *OsTBP2.2*, indicating their strong genetic effects across ecological conditions. Screening the 33 QTLs in the National Rice Data Center gene database (https://www.ricedata.cn/gene/) identified five candidate genes linked to heading date variation. Functional categories of these QTL-associated genes include glyoxalase II (*OsGR2*; *OsGLYR2*), transcription factors (*OsbHLH024*, *OsTBP2.2*), RNA-binding proteins (*OsRRM*), Polycomb Group (PcG) pro-teins (*OsEMF2b*), and nuclear localization proteins (*GW9*).

Based on the gene annotation results of significant intervals (**Table 2**), the key genes associated with rice plant height growth rate are primarily involved in biological processes such as photorespiration, sugar metabolism, cell elongation, and hormone signaling regulation. Among these, *OsGR2* (glyoxalase II) is linked to energy metabo-lism and may enhance early cell elongation by improving photorespiration efficiency. *OsbHLH024*, a transcription factor of the bHLH family, regulates internode development. *OsRRM* (RNA-binding motif protein), associated with sugar transport, supports energy supply during growth. *OsEMF2b*, a Polycomb Group (PcG) protein, influences cytokinin metabolism and internode cell proliferation. *OsTBP2.2*, a transcriptional regulator, is closely related to stem elongation rates. In early rice trials, the growth rate peaks occurred around 26 days after transplanting in both experimental and replicate plots, while late rice experimental plots showed an earlier peak (~22 days), with replicates lagging until 30 days. GWAS analysis revealed that accessions carrying favorable alleles of *OsRRM* and *OsEMF2b* exhibited earlier growth rate peaks (22–25 days) across multiple environments, suggesting these genes accelerate energy allocation and cell elongation, enabling earlier entry into rapid growth phases. Collectively, the identified significant loci and candidate genes establish a core genetic framework for early plant height growth dynamics. This provides critical insights into the molecular mechanisms underlying rice early growth vigor and facilitates marker-assisted selection for improved agronomic traits.

**Table 2:** Candidate genes related to predicting the growth rate of rice plant height and their functional annotations.

Chromosome	Locus	Candidate gene	Functional annotation	Reference gene
1	*qGDR1.1*	*LOC_Os01g39270*	Glyoxylate reductase	*OsGR2; OsGLYR2* (Zhang Z. et al., 2020)
1	*qGDR1.1*	*LOC_Os01g39330*	Basic helix–loop–helix transcription factor	*OsbHLH024* (Alam et al., 2022)
9	*qGDR9.2*	*LOC_Os09g12730*	Spen-like rice gene; RNA-binding protein	*OsRRM* (Chen et al., 2007; Liu et al., 2020)
9	*qGDR9.5*	*LOC_Os09g13630*	Suppressor of Zeste; polycomb group gene	*OsEMF2b* (Wang H. et al., 2022)*; GW9*
9	*qGDR9.6*	*LOC_Os09g13630*	Suppressor of Zeste; polycomb group gene	*OsEMF2b; GW9*
10	*qGDR10.7*	*LOC_Os10g29660*	Transcription factor IID	*OsTBP2.2*

## 4 Discussion

### 4.1 Advantages and limitations of UAV LiDAR dynamic monitoring

This study utilized multi-temporal UAV-based LiDAR point cloud data to achieve high-throughput dynamic monitoring of early rice plant height. Compared to traditional manual measurements or image-based inversion methods, LiDAR offers the ad-vantage of penetrating the canopy to directly extract three-dimensional structural features, enabling continuous and precise extraction of dynamic height changes in both individual plants and populations under unconstrained lighting and background conditions. This technology captures subtle growth differences during early develop-mental stages, providing high-precision phenotypic support for elucidating the relationship between growth rates and genetic regulation.

Compared with UAV-based RGB or multispectral image–derived height estimation methods, LiDAR provides a more direct measurement of vertical structure and is less sensitive to illumination variability, water background interference, and spectral saturation, which are common challenges in paddy rice systems during early growth stages. Image-based approaches generally rely on digital surface model (DSM) reconstruction from structure-from-motion techniques, whose accuracy strongly depends on canopy closure and surface texture (Bendig et al., 2015). As a result, these methods often perform well at mid- to late-growth stages but show reduced reliability during seedling and early tillering phases when canopy coverage is sparse.

In this study, the coefficient of determination (R^2^) for plant height extraction was 0.82, with a root mean square error (RMSE) of 8.75 cm and a relative root mean square error (RMSEr) of 17.7%, indicating that UAV-LiDAR reliably reflects canopy height variations.

However, UAV-LiDAR is susceptible to penetration bias and laser occlusion errors during the seedling stage or sparse canopy phases, leading to underestimation of height—a phenomenon validated in cotton monitoring studies (Xu et al., 2023). Additionally, daily height increments during early growth stages (typically 1–2 cm) may amplify fixed errors during time-series differentiation, causing oscillations in the growth rate curve V(t). To mitigate these errors, a two-stage smoothing strategy was applied before derivative calculation: (1) Savitzky–Golay filtering to suppress high-frequency noise and (2) Piecewise Cubic Hermite Interpolating Polynomial (PCHIP) interpolation to avoid oscillations inherent in traditional polynomial methods. Furthermore, peak identification for V(t)relied on joint screening of peak duration and local waveform morphology rather than single-point thresholds, ensuring that the maximum growth rate time (T_max_) reflects stable physiological growth events rather than noise spikes. Despite potential fluctuations from height measurement errors, statistical reliability of Tmaxextraction was maintained through model constraints and temporal continuity, enabling robust subsequent GWAS analysis. The large divergence in peak growth rate observed between spatial sub-regions (**Figure 9**) indicates that early growth dynamics are highly sensitive to within-field environmental heterogeneity, such as soil nutrient availability, micro-topography, or localized management differences. Nevertheless, UAV-LiDAR has limitations. Coupling between point cloud density, flight altitude, and plant density may cause uneven extraction of canopy surface points, affecting individual plant height accuracy. During the tillering-to-jointing stages, rapid plant growth and wind-induced sway introduce temporal misalignment errors, leading to systematic biases. Additionally, post-processing algorithms (e.g., ground filtering, canopy extraction, and height difference calculation) require parameter optimization based on growth stage characteristics. Future work should integrate high-resolution imagery or multi-sensor data with deep learning to enhance LiDAR point cloud processing for individual plant recognition and growth trait extraction.

It should be noted that GWAS in this study was performed on the timing of maximum growth rate (T_max_) rather than absolute plant height. As a relative temporal trait, T_max_ is less sensitive to systematic height offsets caused by LiDAR penetration bias or early-stage canopy sparsity. Therefore, even when absolute height estimation contains fixed errors, the temporal position of the growth-rate peak remains comparatively robust for genetic association analysis.

### 4.2 Analysis of genetic mechanism of plant height growth rate

This study analyzed early rice plant height growth rates across 211 rice varieties and found that the growth rate distributions in early rice trials, early rice replicates, late rice trials, and late rice replicates all exhibited continuous near-normal distributions.

We found through comparison that the earlier timing of T_max_ in Guangdong rice varieties, particularly early-season types, suggests that local germplasm exhibits faster early-stage growth dynamics than international germplasm. This characteristic likely reflects long-term regional breeding and selection under subtropical double-cropping systems, where rapid early establishment is advantageous for shortening the growth cycle, improving resource capture, and ensuring timely progression to subsequent developmental stages. Interestingly, despite the difference in growth timing, the absence of significant differences in V_max_ indicates that Guangdong and IRRI varieties share similar intrinsic growth capacity once peak growth is reached. This decoupling between growth timing (T_max_) and growth intensity (V_max_) highlights that early vigor in local varieties is mainly expressed through accelerated developmental progression rather than enhanced maximum growth rates.These results demonstrate the importance of considering dynamic growth parameters, rather than single-time-point height measurements, when characterizing early vigor. The ability of UAV-LiDAR–derived time-series phenotypes to resolve subtle differences in growth timing provides valuable insights into genotype-specific growth strategies and offers a robust phenotypic basis for subsequent genetic association analyses targeting early growth regulation.

The maximum growth rate time (T_max_) in late rice experimental plots lagged approximately 8 days behind replicates, potentially due to lower soil nitrogen availability in replicates. Previous studies have shown that nitrogen deficiency delays leaf area expansion, thereby postponing the rapid height growth phase (Yu et al., 2020; Hussain et al., 2022). Since this study’s GWAS was based on multi-environment joint analysis across two seasons, environmental variations in T_max_ were controlled via random effects in the MLM model, preserving statistical power. However, this observation highlights the need for future integration of soil nutrient analysis with gene regulation mechanisms underlying early growth vigor. It is important to emphasize that spatial sub-regions within a single field block do not constitute independent biological replicates. Although these subdivisions provide insight into spatial heterogeneity, they do not increase statistical power or support replicated inference. Accordingly, all genetic interpretations in this study are based on single-year, single-block observations.

A total of five QTLs associated with rice plant height growth rates were detected. ZHANG et al (Zhang Z. et al., 2020). demonstrated that overexpression, single-knockout, and double-knockout mutants of OsGR1and OsGR2exhibited dwarf phenotypes under photorespiration-promoting conditions. ALAM et al (Alam et al., 2022). reported that the *OsbHLH024* mutant (A91) showed significant height differences compared to wild-type (WT) seedlings during the seedling stage, with A91 plants attaining 6.13% greater height during the reproductive phase. LIU et al (Liu et al., 2020). observed that OsRRM mutants exhibited altered mRNA levels of sugar transporter genes, disrupting sugar metabolism and signaling, which in turn affected plant height, flowering time, seed size, and starch synthesis. CHEN et al (Chen et al., 2007). found that ectopic expression of *OsRRM* in transgenic plants caused severe growth defects, including stunted growth and reduced fertility. WANG et al. revealed that RLBphysically interacts with *OsEMF2b*(a Poly-comb Repressive Complex 2 component), localizes to the *OsCKX4* promoter, and elevates *H3K27me3* levels to suppress *OsCKX4* transcription, thereby reducing cytokinin degradation and promoting lateral branching. ZHANG et al (Zhang Y. et al., 2020). generated an *OsTBP2.2T*-DNA insertion mutant (ostbp2.2), which displayed reduced plant height, tiller number, and biomass accumulation in leaves, stems, sheaths, and panicles during flowering and maturity stages. Collectively, these findings confirm the existence of one or more QTLs governing early rice growth rates and suggest that this trait is a complex quantitative characteristic controlled by multiple genes. Given the single-year, single-replicate experimental design, the GWAS results presented here should be interpreted as exploratory associations that highlight candidate genomic regions potentially involved in early growth-rate regulation, rather than as definitive or broadly stable QTLs.

Although spatial plot layout was controlled, soil heterogeneity and unmeasured field variation may still influence early growth dynamics. Because only one biological replicate was available per genotype, such effects could not be statistically separated from genetic effects. Future studies incorporating spatial statistical models and replicated trials are required to address this limitation.

### 4.3 Guiding significance for molecular breeding

The early growth advantage in rice, manifested as phenotypic traits such as rapid height increase during the seedling and tillering stages, forms a critical foundation for subsequent canopy structure development, root system establishment, and yield formation. This study established a quantitative and traceable framework for evaluating early growth vigor by integrating multi-temporal UAV-LiDAR-based height monitoring with genome-wide association studies (GWAS). Although the individual components of the proposed workflow rely on established phenotyping and GWAS techniques, the novelty of this study lies in redefining early growth vigor as a dynamic, time-resolved trait and constructing a phenotyping–genetic analysis pipeline centered on growth-rate timing rather than static measurements. This stage-oriented integration enables genetic dissection of early growth processes that are often overlooked in conventional height-based GWAS studies. Key single nucleotide polymorphism (SNP) loci and candidate genes identified in this study provide potential molecular markers for early plant height growth rate analysis. These markers can be utilized in marker-assisted selection (MAS) to screen elite genotypes exhibiting traits like “rapid post-transplant growth and robust seedling establishment.”

Furthermore, stability analysis of significant loci across diverse environments revealed that certain candidate regions exhibited cross-environment consistency, indicating high genetic robustness. This finding guides future multi-environment GWAS studies and offers theoretical support for breeding high-yielding, early-maturing rice varieties adaptable to fluctuating ecological conditions. Future research could integrate transcriptomic and metabolomic data to elucidate the regulatory pathways of these genes during early growth phases, thereby constructing molecular models for growth regulation and advancing precision breeding strategies.

## 5 Conclusions

1. Based on high-throughput temporal point cloud data from UAV-LiDAR, this study established a dynamic monitoring and phenotypic quantification method for early rice plant height, and combined it with genome-wide association studies (GWAS) to explore the genetic regulatory characteristics of plant height growth rate.
2. UAV-LiDAR enables precise dynamic extraction of early rice plant height, significantly enhancing the timeliness and reliability of phenotypic data acquisition, thus providing a new technical approach for analyzing growth process traits.
3. GWAS using “time of growth rate peak emergence” as a phenotypic indicator revealed multiple significant genomic regions associated with early rice plant height growth. Some candidate genes are involved in cell elongation, hormone signaling, and developmental regulation pathways.
4. The study developed a transferable analytical framework of “LiDAR dynamic monitoring–genetic association,” offering technical support for deciphering the genetic basis of early growth vigor and breeding related rice varieties.
5. Future research should further integrate transcriptomic and metabolomic analyses to validate candidate gene functions, optimize intelligent point cloud recognition and spatiotemporal modeling algorithms, and evaluate the stability and consistency of genetic effects of early growth phenotypes under multi-environment conditions. This will promote the deep integration of UAV remote sensing phenotyping and molecular breeding.

## Data Availability

The datasets presented in this study can be found in online repositories. The names of the repository/repositories and accession number(s) can be found in the article/**Supplementary Material**.
